# 298. Oral vs Intravenous Antibiotics in the Treatment of Periprosthetic Joint Infections

**DOI:** 10.1093/ofid/ofaf695.100

**Published:** 2026-01-11

**Authors:** Angelina Liddy, Neeku Salehi, Eric Smith, Javad Parvizi, Daniel Taupin, Elizabeth Gancher, Brooke Olin, Megan Gorleski, Katherine Belden

**Affiliations:** Thomas Jefferson University Hospital, Philadelphia, Pennsylvania; Rothman Orthopaedics, Philadelphia, Pennsylvania; Sidney Kimmel Medical College of Thomas Jefferson University, Philadelphia, Pennsylvania; Rothman Orthopaedic Institute, Philadelphia, Pennsylvania; Thomas Jefferson University Hospital, Philadelphia, Pennsylvania; Division of Infectious Diseases, Sidney Kimmel Medical College at Thomas Jefferson University, Philadelphia, Pennsylvania; Rothman Orthopaedic Institute, Philadelphia, Pennsylvania; Rothman Orthopaedics, Philadelphia, Pennsylvania; Sidney Kimmel Medical College at Thomas Jefferson University, Philadelphia, Pennsylvania

## Abstract

**Background:**

Historically in the United States, periprosthetic joint infection (PJI) has been managed with intravenous (IV) antibiotics. A number of randomized controlled trials (RCTs) have demonstrated non-inferiority of oral (PO) antibiotics compared to IV therapy in the treatment of bone and joint infections. Our study is a non-inferiority trial comparing PO and IV antibiotic regimens in primary hip and knee PJIs.

**Methods:**

This is an ongoing, multi-center, parallel-group, unblinded, non-inferiority RCT approved by our institution's Institutional Review Board with enrollment beginning December, 2020.

Participants include adults with a primary hip or knee arthroplasty, diagnosed with PJI by International Consensus Meeting criteria, and treated with surgery. Exclusion criteria include prior revision, the need for IV antibiotics for other indications, or a lack of an antibiotic option in either arm.

Patients were randomized to receive either PO or IV antibiotics for six weeks. PO rifampin and follow-on antibiotic suppression was allowed in both arms. The primary study end point is failure at one year by Musculoskeletal Infection Society criteria. Descriptive analysis is provided for the preliminary findings.

**Results:**

Forty-five patients completed one-year follow up with 27 and 18 in the PO and IV groups, respectively. They had a median age of 65 years, 57% were male, 8.8% were of non-white race. Twenty patients (44.4%) were treated for primary hip PJI, and 25 (55%) for primary knee PJI. Twenty-five (55%) underwent a two-stage exchange arthroplasty, 13 (26%) had debridement with implant retention, and 8 (17%) had single-stage exchange arthroplasty. The most commonly treated infections were *Staphylococcus spp*. (n= 21) and culture negative infection (n=12).

There were five treatment failures: two (15.4%) in the IV group and three (12.5%) in the oral group. Of IV patients, 44% (n=8) had a catheter complication.

**Conclusion:**

Preliminary findings in 45 patients who completed one year of follow up suggest that PO antibiotic regimens may be a viable and safe alternative to IV therapy in the treatment of primary hip and knee PJI. Study enrollment is ongoing across multiple centers with a larger patient cohort expected to provide a more definitive assessment of this important clinical question.

**Disclosures:**

All Authors: No reported disclosures

